# Psychotic Risk Associated With Cannabinoid Use: A Case Report of Ekbom-Like Delusional Infestation

**DOI:** 10.7759/cureus.100945

**Published:** 2026-01-06

**Authors:** Miguel Pao Trigo, Joana Cavaco Rogrigues, Bruno Luz, Joaquim Sá Couto, Marco Mota Oliveira

**Affiliations:** 1 Department of Psychiatry, Unidade Local de Saúde do Algarve, Faro, PRT

**Keywords:** acute and transient psychotic disorders, cannabis, delta-9-tetrahydrocannabinol, delusional parasitosis, ekbom syndrome, neuropsychiatry, olanzapine, substance-induced psychosis, tactile hallucinations

## Abstract

Chronic use of high-potency cannabinoids has been consistently associated with an increased risk of psychosis, particularly with early onset, daily use and prolonged exposure. However, atypical somatic presentations may be underrecognised and can create diagnostic challenges in clinical practice.

We reported the case of a 49-year-old man with long-standing daily cannabis use who developed delusional infestation characterised by persistent pruritus, tactile hallucinations and a fixed somatic delusional belief. Dermatological and neurological assessments, routine laboratory investigations, and cranial computed tomography did not reveal abnormalities suggestive of an organic aetiology. Mental status examination demonstrated a well-structured somatic delusion with absent insight. Treatment with olanzapine and psychoeducation was initiated, with gradual symptomatic improvement.

This case report aimed to describe an Ekbom-like somatic presentation within a probable cannabis-induced psychotic disorder and to highlight the diagnostic value of structured clinical assessment, laboratory testing and normal neuroimaging findings in excluding secondary causes. This case underscores the need to consider substance-induced psychosis in patients presenting with delusional infestation, and it reinforces the importance of early recognition, careful differential diagnosis and counselling toward substance cessation to improve prognosis.

## Introduction

The association between cannabinoid use and the development of psychopathology, particularly psychotic symptoms, has been the focus of systematic investigation over recent decades. Also, changes in consumption patterns, sociocultural attitudes toward the substance and the substantial increase in the average potency of available products deserve a higher attention to this problem. Cannabis is currently the most widely used illicit psychoactive substance worldwide, with recent estimates indicating annual prevalence rates exceeding 15% among young adults in several European countries, including Portugal [1-3]. This trend has been accompanied by a marked increase in daily use and consumption of products with high concentrations of delta-9-tetrahydrocannabinol (THC), often containing residual levels of cannabidiol (CBD), representing a significant shift from the chemical profile predominant two to three decades ago [4-6].

Current epidemiological evidence demonstrates a consistent association between regular cannabinoid use (particularly when initiated during adolescence), high frequency of use and high-potency products, increases the risk of developing psychotic symptoms or formal psychotic disorders [7-11]. Multicentre studies such as the European Network of National Schizophrenia Networks Studying Gene-Environment Interactions (EU-GEI) have demonstrated a dose-response relationship between cannabis exposure and risk, estimating that a significant proportion of first-episode psychosis cases in regions with high prevalence of high-potency cannabis use may be attributable to cannabis consumption [10]. Similar findings have been observed in population-based studies in Canada, the United States, Denmark and Israel, particularly following the legalisation or liberalisation of recreational markets [12,13].

Beyond epidemiological observations, controlled studies have demonstrated that THC can induce transient psychotomimetic symptoms in healthy individuals, including thought disorganisation, hallucinations, delusional ideation and perceptual processing disturbances [14-16]. These effects were particularly pronounced in individuals with a family history of psychosis, genetic vulnerability or a prior diagnosis of a psychotic disorder, suggesting clinically relevant gene-environment interactions [14,15].

Understanding of the neurobiological mechanisms underlying the psychotogenic potential of cannabinoids has advanced considerably over the past two decades. THC acts as a partial agonist at cannabinoid receptor type 1 (CB1) receptors, which are densely expressed in structures such as the striatum, prefrontal cortex, hippocampus and amygdala [6,8,14].

Modulation of these receptors interferes with the release of neurotransmitters, including dopamine, glutamate and gamma-aminobutyric acid (GABA). Contemporary neurobiological models suggest that repeated exposure to THC may contribute to striatal dopaminergic dysfunction as a central mechanism in aberrant salience attribution theory and, consequently, in the emergence of positive psychotic symptoms [7,9,14].

The role of CBD is relevant within the pharmacodynamic balance: studies have shown that this non-psychoactive compound may exert anxiolytic, antipsychotic and anti-inflammatory effects, partially modulating the actions of THC. However, most recreational cannabis products currently available contain markedly imbalanced THC:CBD ratios, favouring an increased psychotogenic potential [4,6]. Cannabis-induced psychosis may present with heterogeneous clinical features, including persecutory delusions, auditory or visual perceptual disturbances, formal thought disorder and affective disorders [6,8].

Although persecutory ideation is the most frequently reported, clinical literature documents less common presentations, including somatic, mystical or self-referential delusions [10,11,17]. Among the rare somatic manifestations is delusional infestation (DI), defined by a fixed false belief of being infested with organisms (e.g., insects, parasites, worms), often accompanied by tactile hallucinations, pruritus and behaviours such as body inspection or skin manipulation [18,19]. The syndrome may occur as a primary delusional disorder (also referred to as Ekbom syndrome) or secondary to medical conditions, neurological disease, neurodevelopmental disorders or psychoactive substance use, including cocaine, amphetamines and, more rarely, cannabis.

Differentiating primary psychosis from substance-induced psychosis may be clinically and temporally challenging. Current diagnostic classifications, including the Diagnostic and Statistical Manual of Mental Disorders, Fifth Edition (DSM-5) and the International Classification of Diseases, Eleventh Revision (ICD-11), state that substance-induced psychosis should be considered when symptoms emerge during or shortly after substance use, are clinically significant, and are not better explained by a primary psychotic disorder. However, it is important to emphasise that prolonged exposure to cannabinoids, particularly high-potency formulations, may trigger transient psychotic episodes but also increase the likelihood of progression to a persistent psychotic disorder, including schizophrenia [9,11,18].

In clinical practice, early recognition of this association has direct implications for prognosis, therapeutic approach and relapse prevention. Evidence indicates that continued cannabis use following a psychotic episode increases the risk of recurrence and symptom severity, whereas sustained abstinence is associated with better functional outcomes and a reduced likelihood of relapse [10-12].

This case report aimed to describe an Ekbom-like somatic presentation within a probable cannabis-induced psychotic disorder and to highlight the practical diagnostic value of structured clinical assessment, targeted investigations and normal neuroimaging findings in excluding secondary causes.

## Case presentation

A 49-year-old man living with family members, with no known prior psychiatric history and without regular medical follow-up, was evaluated in the emergency department. He had no documented relevant medical comorbidities and no known family history of psychiatric disorders.

Symptom onset was insidious, with gradual progression over several months. The initial complaints were nonspecific pruritus and cutaneous tingling that increased in frequency and intensity. Over time, he developed a persistent conviction that insects were moving beneath his skin, interpreting the sensory experiences as evidence of infestation. He described intrusive tactile sensations ("like bugs crawling"), intense pruritus, compulsive collection of presumed "evidence" (e.g., loose hairs and textile fibres), prolonged skin inspection using magnification devices, and mild self-injurious behaviour (superficial abrasions from repetitive scratching). Functional impact was significant, with severe sleep disturbance, social withdrawal, reduced daily functioning and family tension.

The patient reported daily cannabis use since approximately 18 years of age, estimating seven to eight joints per day, predominantly dried cannabis flower. Product potency was not quantified. He reported no prolonged abstinence periods in recent years, and he was unable to clearly identify short-term symptom improvement with brief abstinence. He did not report a specific temporal worsening immediately following discrete changes in product type or potency; however, the long-standing pattern reflected sustained high-frequency exposure. He denied alcohol, cocaine, amphetamine, psychedelic, benzodiazepine or opioid use, and denied synthetic cannabinoids (e.g., "Spice," "K2"). A review of medications and over-the-counter substances did not identify a clear alternative explanation for symptoms.

Dermatological assessment did not identify lesions suggestive of true infestation, aside from superficial self-induced abrasions. Neurological examination did not reveal objective sensory or motor deficits. Routine laboratory investigations did not reveal clinically relevant abnormalities. Cranial computed tomography did not reveal structural abnormalities, focal lesions, or findings suggestive of infectious, neoplastic, vascular or neurodegenerative pathology; no acute intracranial abnormalities were identified. These normal neuroimaging findings supported the exclusion of an organic cause of secondary psychosis.

**Figure 1:**
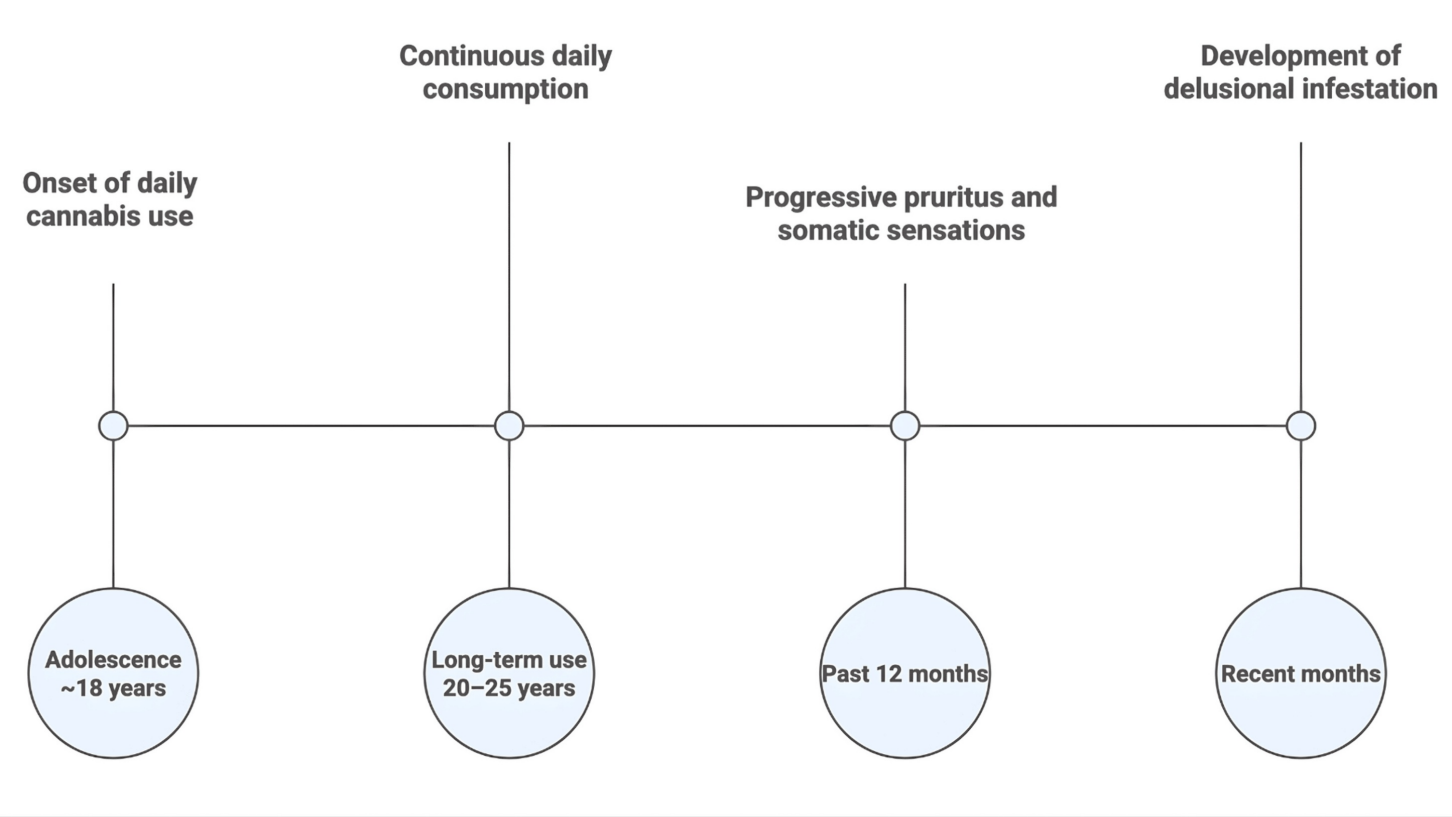
Clinical timeline of cannabis exposure and symptom progression Schematic timeline summarising long-term daily cannabis exposure and the insidious onset and progression of pruritus/tactile symptoms leading to delusional infestation and clinical presentation, followed by initiation of antipsychotic treatment and reported symptomatic improvement. Image Credits: Miguel Pao Trigo

Figure 1 provides a summarised timeline of the patient’s cannabis use history and the progressive development of symptoms leading to clinical presentation.

Table 1 outlines the findings from the patient’s physical and neurological examination, highlighting the absence of organic abnormalities consistent with true infestation or neurological disease.

**Table 1:** Physical examination, neurological examination and complementary investigations Summary of clinical examination and baseline investigations performed to evaluate possible dermatological, neurological or systemic causes of pruritus/tactile symptoms and to exclude organic contributors to secondary psychosis.

Domain	Findings
Dermatological	No pathological lesions suggestive of infestation; superficial self-induced abrasions
Neurological	No objective sensory or motor deficits
Complementary examinations	Full blood count, thyroid function, biochemical panel and cranial CT scan within normal limits

Mental status examination demonstrated a well-structured somatic delusional belief (infestation) with absent insight, prominent somatic hypervigilance, anxious affect and tactile hallucinations, without auditory or visual hallucinations. There was no suicidal ideation. Table 2 summarises the mental status examination, demonstrating clinically significant features.

**Table 2:** Mental status examination Key mental status findings supporting a psychotic presentation with somatic delusional content and absent insight.

Domain	Findings
Mental status examination	Alert and oriented to time, place and person
	Coherent, logical and well-organised speech with no pressured ideation
	Fixed somatic delusional belief (parasitic infestation)
	Tactile hallucinations; no auditory or visual hallucinations
	Anxious affect with prominent somatic hypervigilance
	Absent insight
	No suicidal ideation

The differential diagnosis included primary delusional disorder (somatic type)/primary DI, late-onset schizophrenia-spectrum disorder and secondary psychosis due to medical or neurological conditions. The prominent pruritus and paresthesia-like symptoms also warranted consideration of systemic causes of pruritus and neuropathic sensations. Dermatological examination did not support true infestation. Neurological examination did not demonstrate objective deficits, and routine laboratory investigations did not reveal abnormalities suggestive of systemic, metabolic, infectious or nutritional etiologies. Cranial computed tomography showed no structural pathology that could account for secondary psychosis. Taken together, the absence of objective organic findings supported a functional rather than structural aetiology.

Given the long-standing high-frequency cannabis exposure, the clinical phenomenology and partial improvement following antipsychotic treatment and psychoeducation, a substance-induced psychotic disorder was considered the most likely diagnosis, while acknowledging that alternative primary psychotic disorders cannot be fully excluded in the absence of documented prolonged abstinence and longitudinal course.

A probable diagnosis of substance-induced psychotic disorder associated with cannabis use, presenting with Ekbom-like DI, was established. Treatment with olanzapine 5 mg/day was initiated and later increased to 10 mg/day with good tolerability. Psychoeducation was provided, including discussion of the relationship between cannabis exposure and psychotic risk. Complete cessation of cannabis use was strongly recommended, although abstinence status at follow-up remained uncertain. Over several weeks of treatment, there was a progressive reduction in the frequency and intensity of tactile sensations and loosening of the delusional belief, with decreased anxiety. The patient continued outpatient follow-up after discharge.

## Discussion

Epidemiology and magnitude of risk

Epidemiological evidence demonstrates a consistent association between cannabis use and increased risk of psychosis, showing a dose, frequency and potency-dependent relationship [1-6]. Multicentre studies such as EU-GEI have documented that daily use of cannabis with high THC content substantially increases the likelihood of developing a first-episode psychosis [3,4]. The risk is most strongly associated with early onset of use during adolescence, daily or near-daily consumption, exposure to high-THC products exceeding 12-15% with low CBD content, and the presence of pre-existing genetic vulnerability.

The pattern of use described in this case (daily consumption for decades, no significant abstinence periods and probable use of high-potency products) places the patient within a high-risk epidemiological profile.

Neurobiological mechanisms and models of substance-induced psychosis

**Figure 2:**
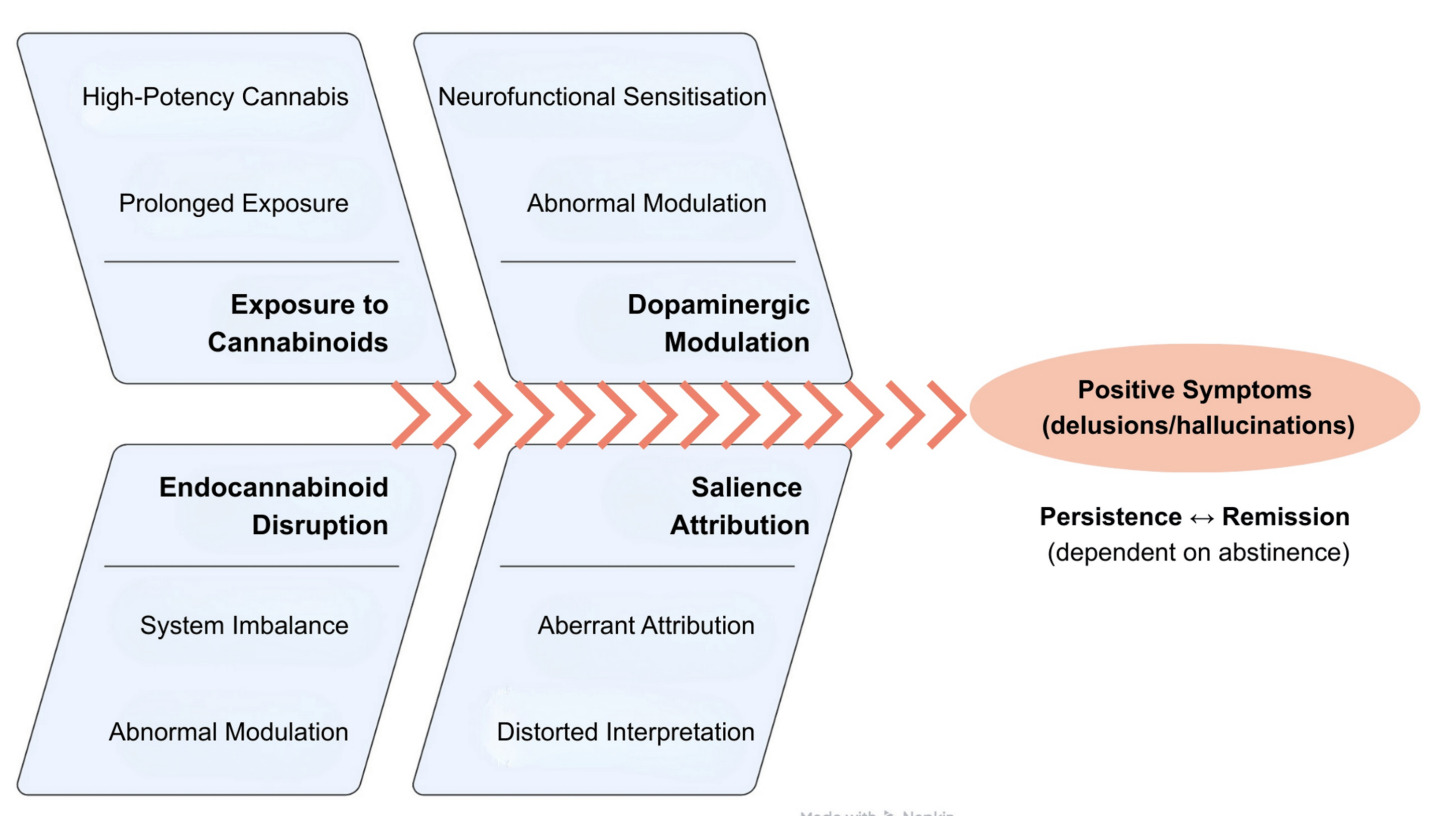
Conceptual model of cannabis-associated psychosis (aberrant salience framework) Schematic illustration of a hypothesis-based pathway by which chronic exposure to high-THC/low-CBD cannabis may contribute to endocannabinoid dysregulation, downstream dopaminergic alterations and aberrant salience attribution, facilitating the emergence and maintenance of structured delusional beliefs. Image Credits: Miguel Pao Trigo. Adapted from contemporary neurobiological models of psychosis and cannabinoid-related dopaminergic dysregulation [6-11]. THC: delta-9-tetrahydrocannabinol; CBD: cannabidiol

THC acts as a partial agonist at CB receptors, modulating the release of neurotransmitters including dopamine, glutamate and GABA [7-10]. This modulation affects key regions involved in cognition, perception and emotion, namely the prefrontal cortex, ventral and dorsal striatum, hippocampus and amygdala. Functional neuroimaging studies demonstrate that exposure to THC may increase striatal dopamine release, a central mechanism within the neurochemical model of aberrant salience. This theory, outlined in Figure 2, proposes that dopaminergic dysregulation leads to inappropriate attribution of significance or meaning to neutral or irrelevant stimuli. When internal sensations or benign stimuli become erroneously salient, structured delusional beliefs may emerge.

This pathological attribution of salience is considered to contribute to the development of psychotic symptoms, particularly in schizophrenia [11-13].

In the present case, the anomalous interpretation of subjective cutaneous sensations (pruritus, tingling, perceived movement under the skin) can be understood as an expression of this mechanism.

Delusional infestation (DI): clinical and phenomenological context

DI, also referred to as Ekbom syndrome, is a subtype of somatic delusion characterised by a fixed false belief of being infested with parasites or living organisms. It may be accompanied by tactile illusions or hallucinations, repetitive checking behaviours and mild self-injurious actions.

Although frequently documented in primary delusional disorders, DI may also occur secondary to neurological disorders, infections, nutritional deficiencies, dementia or exposure to psychoactive substances, including cocaine, amphetamines and cannabinoids [14-18]. This case aligns with secondary DI, with cannabis-induced aetiology considered most likely.

Differential diagnosis

As shown in Table 3, differential diagnosis included possible dermatological, neurological and psychiatric causes.

**Table 3:** Differential diagnosis considered Differential diagnoses considered for delusional infestation with pruritus/tactile symptoms and the clinical reasoning used to support or deprioritise each possibility based on examination, investigations and clinical course.

Diagnosis	Comment	Exclusion/Confirmation
True dermatological infestation	No physical findings	Excluded
Peripheral neuropathy	Neurological examination normal	Excluded
Primary psychosis (schizophrenia)	Possible, but temporal association supports substance-induced psychosis	Deferred
Somatic delusional disorder	Phenotype consistent; medically induced aetiology more likely	Secondary
Substance-induced psychosis	Clear temporal relationship + risk factors + treatment response	Most likely

The temporal association with cannabis use, together with the clinical phenomenology and response to treatment, supported the diagnosis of cannabis-induced psychosis with DI. The absence of insight, the presence of a fixed somatic delusional conviction and the occurrence of tactile hallucinations further strengthened the diagnostic formulation of DI within a substance-induced psychotic presentation.

Treatment: evidence and clinical application

Available evidence suggests that atypical antipsychotics, including risperidone, olanzapine, and, in some cases, amisulpride, are effective in treating psychotic presentations associated with cannabis use and in cases of DI [15-17]. In the present case, olanzapine 5-10 mg/day was well tolerated, with gradual improvement observed and partial loosening of the delusional belief.

Prognosis

Cessation of cannabis use is considered a key prognostic determinant. Current evidence indicates that continued cannabis use triples the risk of relapse after a psychotic episode, while sustained abstinence may gradually reduce the acquired risk, and a subgroup of individuals may progress to persistent psychotic disorder, particularly when genetic or environmental risk factors remain present [5,10,11]. Therefore, this case should be considered clinically high-risk, warranting ongoing follow-up.

Table 4 summarises the main risk factors associated with cannabis-induced psychosis, highlighting how multiple high-risk features converged in this case.

**Table 4:** Risk factors associated with cannabis-related psychosis Summary of established cannabis-related risk factors for psychosis and their relevance to the present case (educational synthesis based on cited literature). THC: delta-9-tetrahydrocannabinol; CBD: cannabidiol

Item	Evidence	Estimated Risk	Key References
Daily cannabis use	Strong association with increased risk of psychosis	High	[1,2,10,12]
Early onset (<21 years)	Increased neurodevelopmental vulnerability and higher incidence of psychosis	High	[1,11,13]
High-potency products (>12-15% THC)	Robust association with first-episode psychosis and symptom severity	High	[2,19]
Low CBD content	Reduced protective modulation of THC effects	Moderate-High	[6,19]
Prolonged duration (>5 years)	Increased likelihood of dopaminergic sensitisation	High	[6,9]
Family history of psychosis	Amplifies cannabis-related psychotic risk via gene-environment interaction	High	[11,18]
Continued use after a psychotic episode	Associated with relapse and poorer functional outcomes	Very high	[10,12]
Sustained abstinence (>6-9 months)	Significant reduction in relapse risk and possible partial risk normalisation	Protective	[10,12]

## Conclusions

﻿﻿Regular use of high-potency cannabis has been consistently associated in epidemiological and clinical studies with an increased risk of psychotic symptoms and psychotic disorders, particularly when initiated in adolescence and maintained on a daily basis. DI may represent an atypical somatic presentation within substance-induced psychosis and requires a structured differential diagnosis to exclude dermatological, neurological, metabolic and infectious causes. Sustained abstinence from cannabis is an important prognostic factor; continued use after a psychotic episode has been associated with higher relapse risk and poorer outcomes, whereas abstinence is associated with improved prognosis. Second-generation antipsychotics, including olanzapine, may be clinically helpful, but psychosocial interventions and relapse-prevention strategies remain essential.

This case illustrates an Ekbom-like presentation of DI in the context of long-standing high-frequency cannabis use. It highlights the clinical importance of considering substance-induced psychosis when patients present with atypical somatic delusions and prominent pruritus, and it underscores the diagnostic value of structured clinical assessment, targeted investigations and normal neuroimaging findings in excluding secondary causes. Because this is a single case without documented prolonged abstinence and without standardised symptom rating scales or follow-up neuroimaging, causal inference is limited, and the observations should be interpreted as hypothesis-generating rather than generalisable. Early recognition, appropriate antipsychotic treatment and clear counselling toward sustained cannabis cessation are practical steps that may reduce morbidity and relapse risk. Future research should further characterise somatic presentations of cannabis-associated psychosis and identify clinical and biological predictors of persistence versus remission.
